# Analysis of Schwann Cell Migration and Axon Regeneration Following Nerve Injury in the Sciatic Nerve Bridge

**DOI:** 10.3389/fnmol.2019.00308

**Published:** 2019-12-10

**Authors:** Bing Chen, Quan Chen, David B. Parkinson, Xin-peng Dun

**Affiliations:** ^1^Department of Neurology, The Affiliated Huai’an No.1 People’s Hospital of Nanjing Medical University, Huai’an, China; ^2^Faculty of Health: Medicine, Dentistry and Human Sciences, Plymouth University, Plymouth, United Kingdom

**Keywords:** peripheral nerve, injury, nerve bridge, Schwann cell, migration, axon regeneration

## Abstract

While it is proposed that interaction between Schwann cells and axons is key for successful nerve regeneration, the behavior of Schwann cells migrating into a nerve gap following a transection injury and how migrating Schwann cells interact with regenerating axons within the nerve bridge has not been studied in detail. In this study, we combine the use of our whole-mount sciatic nerve staining with the use of a proteolipid protein-green fluorescent protein (PLP-GFP) mouse model to mark Schwann cells and have examined the behavior of migrating Schwann cells and regenerating axons in the sciatic nerve gap following a nerve transection injury. We show here that Schwann cell migration from both nerve stumps starts later than the regrowth of axons from the proximal nerve stump. The first migrating Schwann cells are only observed 4 days following mouse sciatic nerve transection injury. Schwann cells migrating from the proximal nerve stump overtake regenerating axons on day 5 and form Schwann cell cords within the nerve bridge by 7 days post-transection injury. Regenerating axons begin to attach to migrating Schwann cells on day 6 and then follow their trajectory navigating across the nerve gap. We also observe that Schwann cell cords in the nerve bridge are not wide enough to guide all the regenerating axons across the nerve bridge, resulting in regenerating axons growing along the outside of both proximal and distal nerve stumps. From this analysis, we demonstrate that Schwann cells play a crucial role in controlling the directionality and speed of axon regeneration across the nerve gap. We also demonstrate that the use of the PLP-GFP mouse model labeling Schwann cells together with the whole sciatic nerve axon staining technique is a useful research model to study the process of peripheral nerve regeneration.

## Introduction

Peripheral nerve injuries are common in both civil and military environments and are primarily transection injuries (Deumens et al., 2010; Ray and Mackinnon, 2010; Daly et al., 2012). Transection injuries can occur during motor vehicle accidents, sports activities, surgery or other forms of penetrating trauma. Damage to the peripheral nervous system typically leads to the development of neuropathic pain and life-long loss of motor and sensory function. The effective treatment of peripheral nerve transection injuries is a clinically significant and challenging area and further research is required in order to improve the outcome of peripheral nerve repair (Moore et al., 2009; Daly et al., 2012).

Accumulating evidence indicates that following injury, regenerating axons are unable to cross a peripheral nerve gap without Schwann cell guidance at their migrating growth front (Torigoe et al., 1996; Parrinello et al., 2010; Webber et al., 2011; Rosenberg et al., 2014; Cattin et al., 2015; Dun and Parkinson, 2015). Using an anti-mitotic agent (mitomycin C) to prevent Schwann cell division, Hall (1986) showed that inhibition of Schwann cell proliferation and migration after mouse sciatic nerve transection injury significantly impeded axon regeneration. In the zebrafish motor axon transection injury model, regenerating axons lost their direction and traveled along ectopic trajectories in the nerve bridge when Schwann cells were genetically ablated (Rosenberg et al., 2014). Additionally, in elegant experiments using a vascular endothelial growth factor (VEGF) bound bead to misdirect both blood vessel regeneration and Schwann cell migration in the rat sciatic nerve gap, regenerating axons followed the path of ectopic migrating Schwann cells and left the nerve bridge (Cattin et al., 2015). These findings showed that Schwann cells play a pivotal role in controlling the directionality of regenerating axons in the peripheral nerve gap. Thus, understanding how Schwann cells direct axon regeneration in a nerve gap, and the relative chronology of Schwann cell migration and axonal growth, is vital in order to develop new therapeutic strategies for boosting peripheral nerve repair. To date, how migrating Schwann cells interact with regenerating axons in the peripheral nerve bridge during regeneration has not been fully studied, largely due to the inability to visualize Schwann cell-axon interaction *in vivo*, and this is the purpose of this current study.

Recently, we developed a whole-mount staining method to study the pattern of axon regeneration in the nerve gap following mouse sciatic nerve transection injury (Dun and Parkinson, 2015). The use of this technique has allowed us to precisely map patterns of axon regeneration within the nerve bridge. In this study, we apply the whole-mount staining technique on nerve bridge tissue in the proteolipid protein-green fluorescent protein (PLP-GFP) mouse strain (Mallon et al., 2002), which expresses GFP in Schwann cells of the peripheral nerves driven by the mouse myelin PLP gene promoter. We examined *in vivo* axon regeneration, Schwann cell migration and Schwann cell-axon interactions in the mouse sciatic nerve bridge. Combining our whole-mount staining method with the PLP-GFP mouse model, we demonstrate that Schwann cells play a crucial role in guiding axon regeneration across a nerve gap after peripheral nerve transection. We also demonstrate that the use of the PLP-GFP mouse model labeling Schwann cells together with the whole sciatic nerve axon staining technique could provide a useful research model to study the process of peripheral nerve regeneration.

## Materials and Methods

### Animal Husbandry and Peripheral Nerve Surgery

The PLP-GFP mouse transgenic strain was used in this study (Mallon et al., 2002). Originally made to label oligodendrocytes in the central nervous system driven GFP expression by the mouse myelin PLP gene promoter, the PLP-GFP mice also express cytoplasmic GFP in both myelinating and non-myelinating Schwann cells of the peripheral nerves (Mallon et al., 2002; Carr et al., 2017; Stierli et al., 2018; Dun et al., 2019). All work involving animals was performed according to Home Office regulation under the UK Animals (Scientific Procedures) Act 1986. Ethical approval for all experiments was granted by Plymouth University Animal Welfare and Ethical Review Board. For sciatic nerve surgery, equal numbers of 2-month-old male and female mice were anesthetized with isoflurane, the right sciatic nerve was exposed and transected at approximately 0.5 cm proximal to the sciatic nerve trifurcation site and no re-anastomosis of the severed nerve was performed. This approach allowed analysis of axon pathfinding and Schwann cell migration within the nerve bridge that forms between the retracted proximal and distal nerve stumps. Following nerve transection surgery, the overlying muscle was sutured and the skin was closed with an Autoclip applier. All animals undergoing surgery were given appropriate post-operative analgesia and monitored daily. At the indicated time points post-surgery for each experiment described, animals were euthanased humanely by CO_2_ in accordance with UK Home Office regulations.

### Whole-Mount Staining

At the described time points following surgery, nerves were dissected out together with surrounding muscle to ensure the nerve bridge structure remained fully intact. Nerves together with surrounding muscles were fixed in 4% paraformaldehyde for 5 h at 4°C. Following fixation and PBS wash, surrounding muscle tissue was carefully removed in PBS using a dissecting microscope. Nerves were then washed in PTX (1% Triton X-100; Sigma, T9284) in PBS three times for 10 min each wash and then incubated with blocking solution [10% fetal bovine serum (FBS) in PTX] overnight at 4°C. The following day, nerves were transferred into primary antibodies in PTX containing 10% FBS and incubated for 72 h at 4°C with gentle rocking. The primary antibody used for the experiments is an anti-neurofilament heavy chain chicken polyclonal (1:100, Abcam, ab4680, immunogen, cow full-length intermediate filaments). After the incubation, nerves were washed three times with PTX for 15 min each wash, followed by washing in PTX for 6 h at room temperature, with a change of PTX every hour. Alexa Fluor 568 dye conjugated anti-chicken secondary antibody (1:500, Invitrogen, Carlsbad, CA, USA) was diluted in PTX containing 10% FBS, and incubated with the nerve preparation for 48 h at 4°C with gentle rocking. Nerves were then washed in PTX three times for 15 min each, followed by washing in PTX for 6 h at room temperature, changing the PTX each hour, and then washed overnight without changing PTX, at 4°C. Due to the nature of long time antibody incubation of this whole nerve staining method, buffers were filtered through 0.45 μm filters to maintain sterile conditions. Nerves were cleared sequentially with 25%, 50%, 75% (v/v) glycerol (Sigma, G6279) in PBS between 12–24 h for each glycerol concentration. Following clearing, nerves were mounted in CitiFluor (Agar Scientific, R1320) for confocal microscopy and image acquisition.

### Imaging

Images were obtained with a Zeiss LSM510 confocal microscope. Several Z-series were captured, covering the entire field of interest. The individual series were then flattened into a single image for each location and combined into one image using Adobe Photoshop software (Adobe Systems, San Jose, CA, USA).

### Data Quantification and Statistical Analysis

The bridge length, the distance of leading Schwann cells from the nerve ends, the area of migrating Schwann cells in the nerve bridge and the speed of axonal growth were measured using Image-J following image acquisition. The bridge length was measured as the distance between the two nerve ends (indicated by two dashed lines in all Figures). The distance of leading Schwann cells on day 4, day 5 and day 6 was measured as the distance from the nerve ends to the leading migrating Schwann cells. To calculate the average speed of axonal growth on day 6 and day 7, the distance of leading axons from the proximal nerve end was measured on day 5 and day 7. The average speed of axonal growth (μm/day) was calculated using the distance difference between day 7 and day 5 divided by 2 (day 6 and day 7). Statistical analysis was carried out using the student’s *t*-test. Data were presented as Mean ± SEM in the article, *n* = 4 for each timepoint.

## Results

### Axon Regeneration Is Ahead of Schwann Cell Migration in the Proximal Nerve Stump

Sciatic nerve transection is the most frequently used research model for studying peripheral nerve regeneration in rodents (Dun and Parkinson, 2018). Previous studies have confirmed that Schwann cells of the peripheral nerves express high levels of GFP in the PLP-GFP mouse model, which allows us to accurately visualize Schwann cell behavior and migration following sciatic nerve transection (Mallon et al., 2002; Cattin et al., 2015; Carr et al., 2017; Stierli et al., 2018; Dun et al., 2019). In our experiments, sciatic nerve transection generated a nerve bridge gap of 1.62 ± 0.29 mm, allowing us to observe the axon extension from the proximal nerve stump together with Schwann cell migration from both proximal and distal nerve stumps.

**Figure 1:**
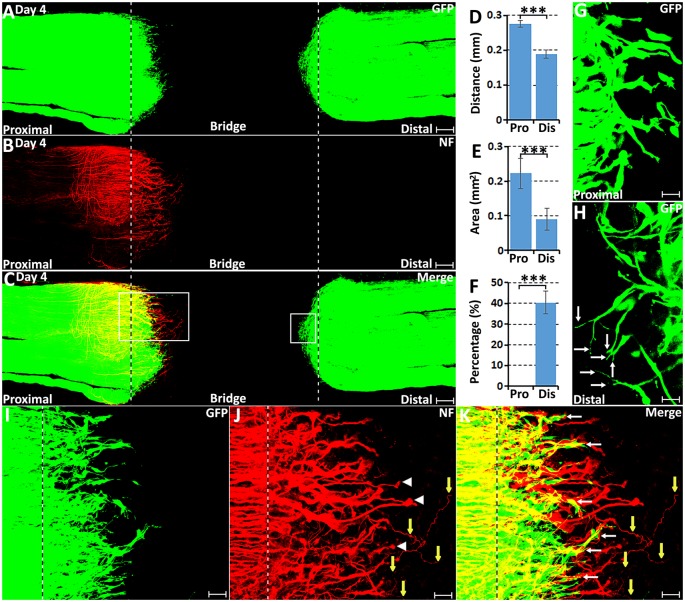
Axon regeneration and Schwann cell migration in the sciatic nerve bridge at 4 days post-injury. **(A–C)** Whole nerve preparation at 4 days post-injury shows regenerating axons and migrating Schwann cells in the nerve bridge of proteolipid protein-green fluorescent protein (PLP-GFP) mice. **(D)** Distances of the leading migrating Schwann cells from the proximal (Pro) stump and the distal (Dis) nerve ends. **(E)** The area of migrating Schwann cells in the proximal (Pro) part and the distal (Dis) part of the nerve bridge. **(F)** Percentage of leading Schwann cells having two or three leading processes. **(G)** On day 4, leading Schwann cells migrating from the proximal stump appear to have a single migrating process, whereas** (H)** about 40% leading Schwann cells migrating from the distal nerve stump show two or three leading processes (indicated by arrows). **(I–K)** Higher magnification images from the box area of proximal nerve stump in **(C)** showing regenerating axons proceeding in front of migrating Schwann cells in the proximal nerve stump at 4 days post-injury. Regenerating axons form axon bundles and appear to have ball shapes at their tips (indicated by white arrow heads in **J**). White arrows in **(K)** indicate Schwann cells apparently migrating along the regrowing axons. Yellow arrows in **(J,K)** show several single regenerating axons growing in a random direction within the nerve bridge. The sites of transection for both the proximal and distal nerve stumps are indicated by dashed lines in each image. ***P < 0.001. Scale bars in **(A–C)** 150 μm. Scale bars in **(G,H)** 25 μm. Scale bars in **(I–K)** 50 μm.

In agreement with previous findings using the S100 marker to identify migrating Schwann cells (Parrinello et al., 2010; Cattin et al., 2015), we also observed that GFP positive Schwann cells start to migrate into the nerve bridge from both proximal and distal nerve ends at 4 days post-transection in our whole-mount PLP-GFP sciatic nerve preparations (Figure 1A). In both the proximal and the distal nerve stumps on day 4, a few Schwann cells have migrated past the transection site (indicated by dashed lines in Figures 1A,I) and into the nerve bridge. While Schwann cells are migrating at this timepoint, whole-mount nerve neurofilament staining showed that regenerating axons in the proximal end are clearly proceeding in front of migrating Schwann cells on day 4 (Figures 1B,C). At this early timepoint, there are no Schwann cells associated with the front wave of regenerating axons (Figures 1C,K). On day 4 in the proximal nerve stump, Schwann cells appear to use regenerating axons as a substrate to migrate toward the nerve bridge (indicated by white arrows in Figure 1K). On day 4 in the distal nerve stump, about 40% of the leading Schwann cells have two or three leading processes (Figure 1F), indicating that they are pioneer cells and are seemingly responsible for detecting environmental signals and searching for a substrate upon which to migrate (Figure 1H). In contrast, leading Schwann cells from the proximal nerve do not have several processes at 4 days, perhaps because they are using regenerating axons as a substrate to migrate (Figure 1G). On day 4, distances of the leading migrating Schwann cells were 275.42 ± 10.1 μm from the proximal stump and 189.79 ± 11.96 μm from the distal nerve stump (Figure 1D). The area of Schwann cells migrating into the nerve bridge from the proximal stump is bigger than 0.2 mm^2^ but the area of Schwann cells migrating into the nerve bridge from the distal nerve stump is smaller than 0.1 mm^2^ (Figure 1E). The distance and area difference between the proximal and the distal nerve stump indicate that Schwann cells from the proximal nerve stump migrate faster than those from the distal nerve stump on day 4, potentially due to the fact that Schwann cells in the proximal nerve stump are using axons as a substrate upon which to migrate at this time.

Previously using whole-mount staining in C57BL/6 mice, we showed that 5 or 6 regenerating axons formed axon bundles resulting in seemingly large diameter axons observed at this stage of regeneration, a ball shape is often formed at the tips of these axon bundles (Dun and Parkinson, 2015). In the PLP-GFP mice, we also observed the ball shape at the tips of regenerating axons (Figure 1J). Occasionally, three to five single axons could be observed extending further into the nerve bridge but were not facing towards the distal nerve stump (Figures 1J,K, indicated by yellow arrows). These observations indicate that axon regeneration occurs significantly earlier than Schwann cell migration but axons appear to lack directionality at this early stage of regeneration.

**Figure 2:**
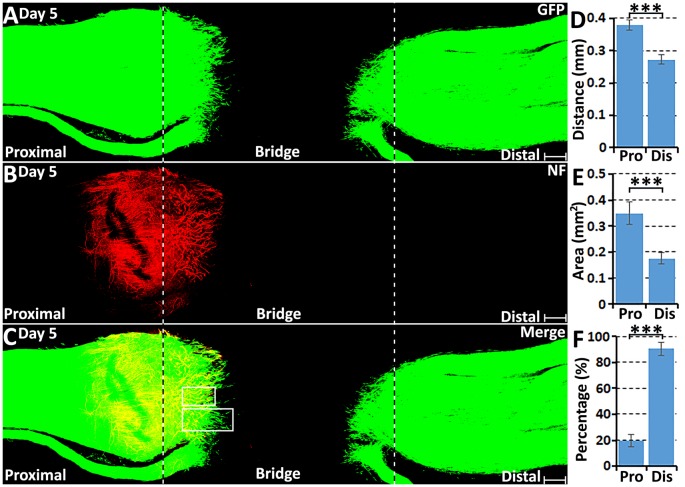
Axon regeneration and Schwann cell migration in the sciatic nerve bridge on day 5 post-injury. **(A–C)** Whole nerve preparation on day 5 shows regenerating axons and migrating Schwann cells in the nerve bridge of PLP-GFP mice. The cut ends of both proximal and distal nerves are indicated by dashed lines. **(D)** Distances of the leading migrating Schwann cells from the proximal (Pro) stump and the distal (Dis) nerve ends. **(E)** The area of migrating Schwann cells in the proximal (Pro) part and the distal (Dis) part of the nerve bridge. **(F)** Percentage of leading Schwann cells having two or three leading processes. ***P < 0.001. Scale bars in **(A–C)** 150 μm.

**Figure 3:**
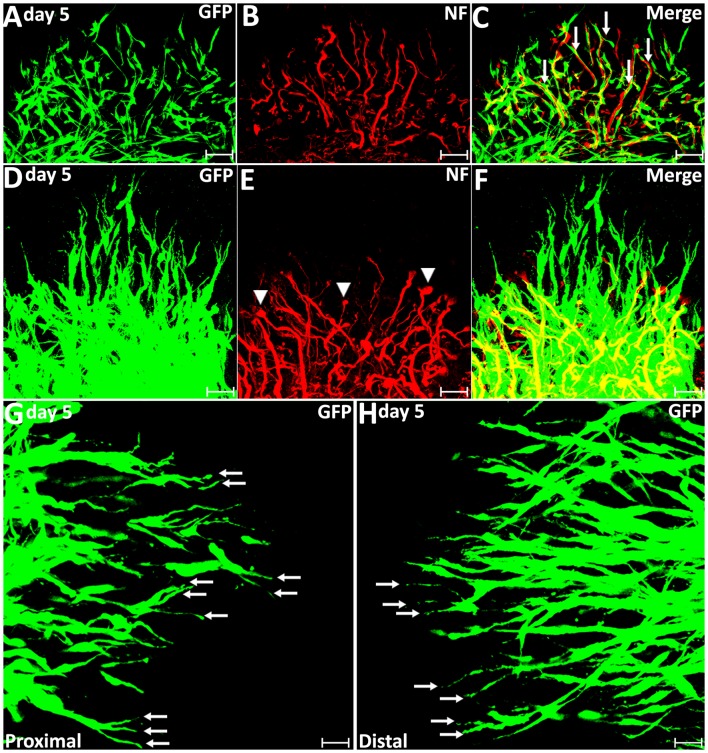
Axon regeneration and Schwann cell migration in the sciatic nerve bridge on day 5 post-injury. **(A–C)** On day 5 in the proximal nerve stump, Schwann cells appear to use regenerating axons as a substrate to migrate upon, indicated by white arrows in **(C)**. **(D–F)** A few migrating Schwann cells start to proceed ahead of the regenerating axon front from day 5 post-injury in the proximal nerve stump. Arrow heads in **(E)** show the ball shape at the tips of regenerating axons.** (G,H)** On day 5, leading Schwann cells from both proximal and distal nerve stumps show two or three leading processes (indicated by arrows). Chain Schwann cell migration is easily visible from the distal nerve stump on day 5. Scale bars in **(A–F)** 40 μm. Scale bars in **(G–H)** 25 μm.

From day 5, robust Schwann cell migration into the nerve bridge was seen from both nerve stumps (Figures 2A–C). On day 5, distances of the furthest leading Schwann cells from the cut sites are 379.08 ± 16.73 μm from the proximal nerve stump and 272.97 ± 14.68 μm from the distal nerve stump (Figure 2D). The area of Schwann cells migrating into the nerve bridge was more than 0.3 mm^2^ from the proximal stump and lesser than 0.2 mm^2^ from the distal nerve stump (Figure 2E). Similarly as for the migration rates for day 4, the difference in the migration distances and area between the proximal stump and the distal nerve stump may result from Schwann cells in the proximal nerve stump using axons as a substrate to migrate upon. On day 5 post-injury, migrating Schwann cells in the proximal nerve end still could be observed using regenerating axons as a substrate upon which to migrate (Figures 3A–C), however, a few migrating Schwann cells begin to proceed in front of the regenerating axons on day 5 (Figures 3D–F). Interestingly, about 20% leading Schwann cells in the proximal nerve stump start to show two or more processes once they migrate past the front of regenerating axons (Figures 2F, 3G), indicating that they have now apparently become pioneer cells and are starting to search for a new substrate to migrate upon. On day 5 in the distal nerve stump, more than 80% leading Schwann cells show two or three leading processes (Figures 2F, 3H). From our analysis, before day 5 it appears that Schwann cells are following axons from the proximal nerve stump; following day 5, Schwann cells overtake regenerating axons and proceed in front of the regrowing axon front.

### Migrating Schwann Cells, Leaders to Direct Regenerating Axons Across the Nerve Gap

**Figure 4:**
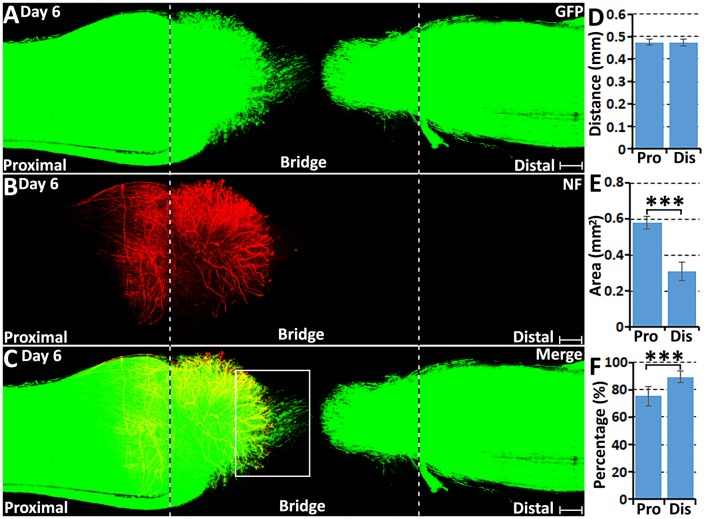
Axon regeneration and Schwann cell migration in the sciatic nerve bridge at 6 days post-injury. **(A–C)** Whole nerve preparation on day 6 shows regenerating axons and migrating Schwann cells in the nerve bridge of PLP-GFP mice. The cut ends of both proximal and distal nerves are indicated by dashed lines. **(D)** Distances of the leading migrating Schwann cells from the proximal (Pro) stump and the distal (Dis) nerve ends. **(E)** The area of migrating Schwann cells in the proximal (Pro) part and the distal (Dis) part of the nerve bridge. **(F)** Percentage of leading Schwann cells having two or three leading processes. ***P < 0.001. Scale bars in **(A–C)** 150 μm.

At 6 days following transection, more migrating Schwann cells were observed within the nerve bridge (Figure 4A), but an area free of Schwann cells was still present in the middle of the nerve bridge between migrating cells from both nerve stumps (Figures 4A–C). Distances of the leading migrating Schwann cells were 474.67 ± 13.56 μm from the proximal stump and 473.67 ± 14.78 μm from the distal nerve stump (Figure 4D). This measurement showed that the distance of the leading migrating Schwann cells in the proximal nerve stump is similar to the distance of the leading migrating Schwann cells from the distal nerve stump on day 6. As indicated by generating several leading processes of leading migrating Schwann cells in the proximal nerve stump once they localize in front of regenerating axons from day 5 onwards, the searching for a new substrate to migrate upon may potentially slow down the migration of Schwann cells in the proximal nerve stump between day 5 and day 6. The area of Schwann cells in the nerve bridge from proximal nerve stump on day 6 nearly reaches to 0.6 mm^2^ but the area of Schwann cells from the distal nerve stump is only 0.3 mm^2^ due to regenerating axons provide a much wider substrate for Schwann cell migration on day 4 and day 5 (Figure 4E).

**Figure 5:**
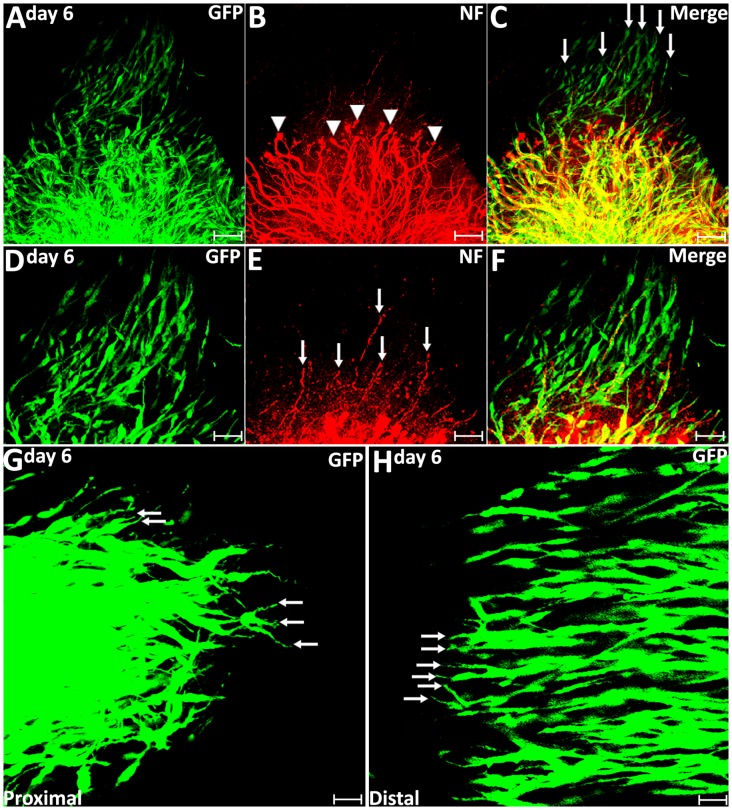
Axon regeneration and Schwann cell migration in the sciatic nerve bridge at 6 days post-injury. **(A–C)** More migrating Schwann cells localize in front of regenerating axon front on day 6 in the proximal nerve stump. Arrow heads in **(B)** indicate the ball shape at the tips of axon bundles. Arrows in **(C)** indicate migrating Schwann cells localized in front of regenerating axons. **(D–F)** Single regenerating axons follow Schwann cell chains on day 6 post-injury in the proximal nerve end. Arrows in **(E)** indicate single axons following the migrating Schwann cell chains. **(G,H)** On day 6, leading Schwann cells from both proximal and distal nerve stumps still show two or three leading processes (indicated by arrows) with chain Schwann cell migration visible in both the proximal stump and the distal nerve stump. Scale bars in **(A–C)** 75 μm. Scale bars in **(D–H)** 50 μm. Scale bars in **(G–H)** 25 μm.

On day 6 post-injury, more than 70% pioneer migrating Schwann cells from the proximal stump and more than 80% pioneer migrating Schwann cells from the distal nerve stump show two or three leading processes (Figures 4F, 5G,H). Following the pioneer cells, Schwann cells attach to each other and form a chain to migrate towards the middle of the nerve bridge (Figures 5G,H). Thus, *in vivo* Schwann cell migration after peripheral nerve transection injury appears to represent classic cell chain migration behavior with the leading cells guiding the followers and forming a chain of migrating cells. Interestingly on day 6, regenerating axons start to change their morphology when there are many migrating Schwann cells in front of regenerating axons (Figures 5A–F). Axon bundles lose their typical ball shape at their tips and single axons start to emerge from axon bundles (Figures 5B,E). Single regenerating axons can be seen to apparently follow the Schwann cell chains and elongate toward the distal nerve stump (Figures 5D–F). This indicates that the rapid single axon growth is seemingly induced by the presence of migrating Schwann cells ahead of the axon front.

**Figure 6:**
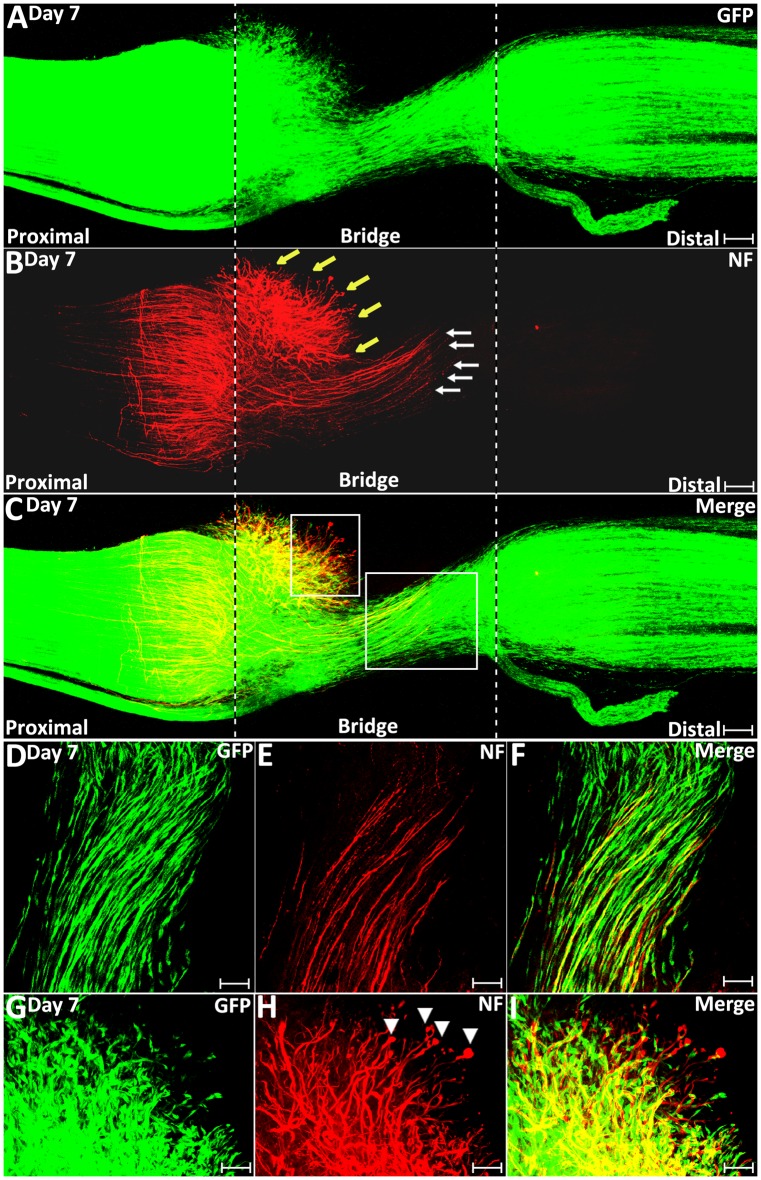
Schwann cell cord formation and axon-Schwann cell interaction in the nerve bridge on day 7 post-injury. **(A–C)** Whole nerve preparation on day 7 post-transection shows regenerating axons and Schwann cell cords in the nerve bridge of PLP-GFP mice. Two populations of regenerating axons (yellow vs. white arrows in **B**) are distinguishable in the nerve bridge at this timepoint. The cut ends of both proximal and distal nerves are indicated by dashed lines. **(D–F)** A population of regenerating axons (indicated by white arrows in **B** and the area of the larger box in C) follow Schwann cell cords and cross the nerve bridge. **(G–I)** A population of regenerating axons (indicated by yellow arrows in **B** and the area of the small box in C) lack Schwann cell guidance in front on day 7 in the nerve bridge. Scale bars in **(A–C)** 150 μm. Scale bars in **(D–F)** 50 μm. Scale bars in **(G–I)** 30 μm.

**Figure 7:**
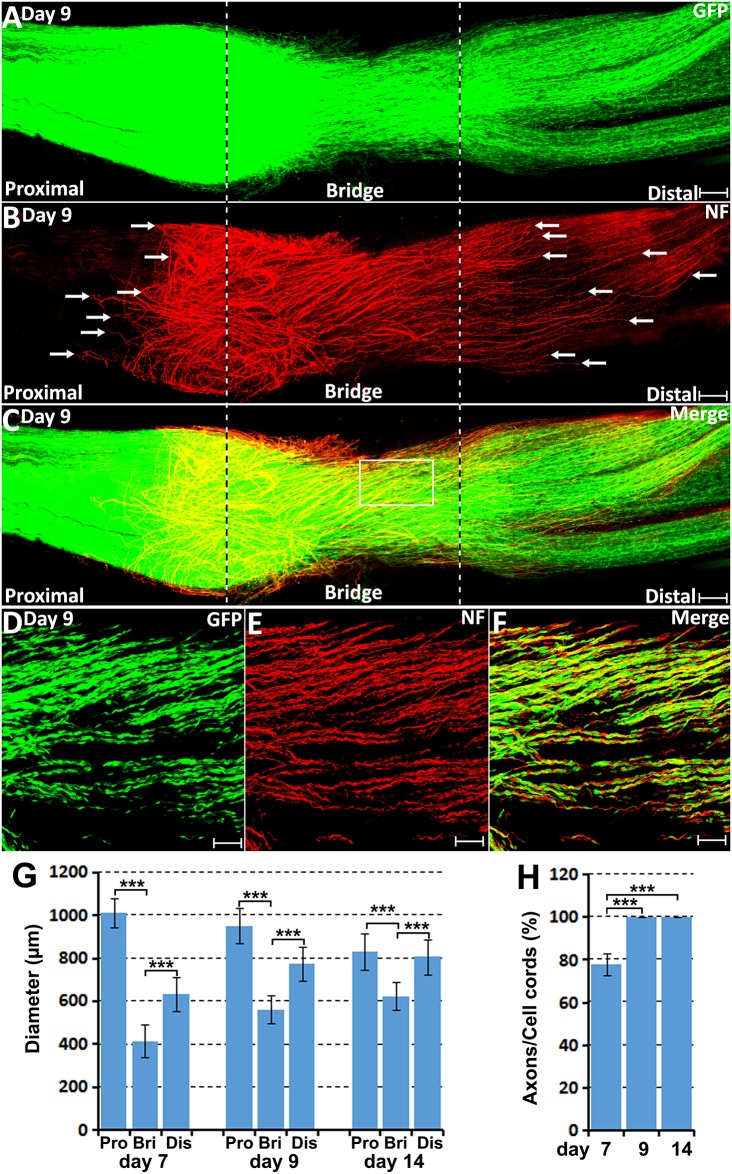
Axon-Schwann cell interactions in the nerve bridge on day 9 in the PLP-GFP mouse. **(A–C)** Whole nerve preparation on 9 days post-injury shows axon-Schwann cell interactions in the nerve bridge. Misdirected axons in the proximal nerve stump were observed growing back along the surface of proximal nerve stump (indicated in the proximal nerve stump by arrows in **B**). Misdirected axons in the distal nerve stump start also to grow along the surface of the distal nerve stump (indicated in the distal nerve stump by arrows in **B**). The cut ends of both proximal and distal nerves are indicated by dash lines. **(D–F)** Several regenerating axons associate with each Schwann cell cord in the nerve bridge on day 9 post-injury. **(G)** The diameter of the proximal nerve end, the diameter of Schwann cell cords in the nerve bridge and the diameter of the distal nerve end on day 7, 9 and 14. **(H)** The percentage of Schwann cell cords associated with regenerating axons in the nerve bridge on day 7, 9 and 14. ***P < 0.001. Scale bars in **(A–C)** 150 μm. Scale bars in **(D–F)** 100 μm.

On day 7 post-transection, migrating Schwann cells from both the proximal stump and the distal nerve stump have mixed in the middle of the nerve bridge and Schwann cell cords have formed to direct axons regenerating towards the distal nerve stump (Figures 6A–C). Schwann cells within the cords can be seen to attach to each other and form longitudinal chains connecting the proximal and distal nerve stumps (Figures 6D–F). Single regenerating axons inside the Schwann cell cords could be observed attaching to the Schwann cell cords and elongating towards the distal nerve stump (Figures 6D–F). At 7 days post-injury, about 78% of Schwann cell cords are associated with a single regenerating axon in the proximal part of the nerve bridge (Figures 6D–F, 7H). On day 7, we observed that a subset of axons inside the Schwann cell cords (indicated by white arrows in Figure 6B) have regenerated much more rapidly than axons (indicated by yellow arrows in Figure 6B), which lack Schwann cell guidance at their front (Figure 6C). As a measure of how the association between Schwann cells with regenerating axons accelerates axonal regeneration, we have measured the average speed of axons growing inside the Schwann cell cord on day 6 and day 7 as 433.1 ± 32.1 μm/day; this is in contrast, the speed of non-Schwann cell-associated axons, which is only 85.7 ± 9.2 μm/day.

**Figure 8:**
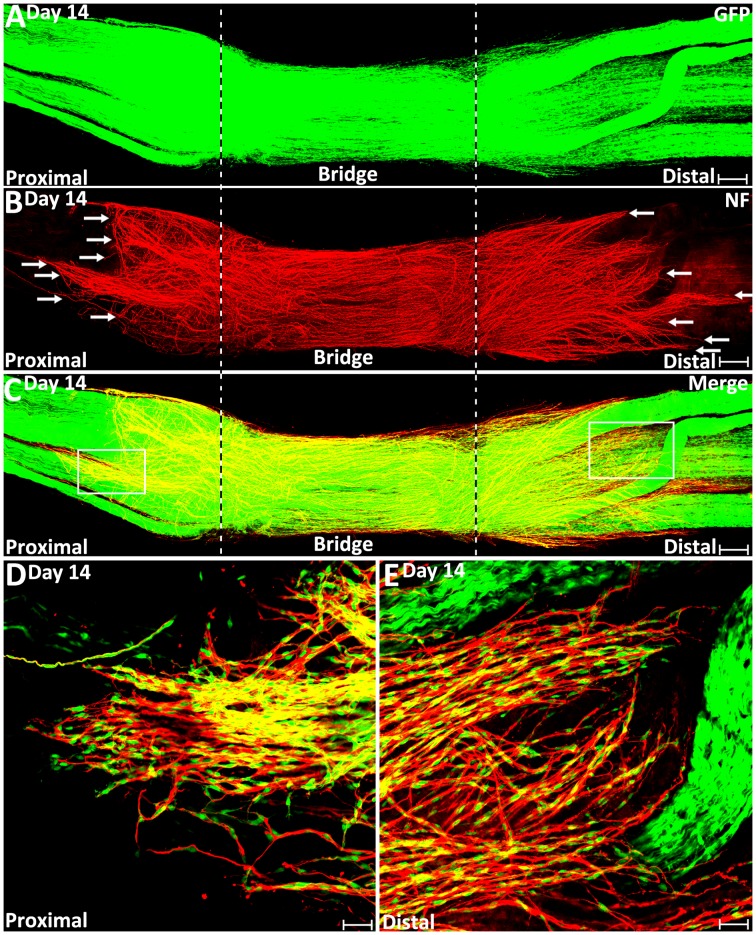
Misdirected axons around the nerve bridge on day 14 post-injury in the PLP-GFP mice. **(A–C)** Whole nerve preparation on day 14 post-injury shows regenerating axons and Schwann cells around the nerve bridge. There are more misdirected axons on the surface of both proximal and distal nerve stumps on day 14 than on day 9 (indicated by arrows). The cut ends of both proximal and distal nerve are indicated by dashed lines.** (D)** Higher magnification image from the box area of proximal nerve stump in **(C)** shows misdirected axons growing along the surface of the proximal nerve stump. **(E)** Higher magnification image from the box area of distal nerve stump in **(C)** shows misdirected axons growing along the surface of the distal nerve stump. Scale bars in **(A–C)** 150 μm. Scale bars in **(D,E)** 50 μm.

On day 9 and day 14 post-injury, more Schwann cells and regenerating axons could be observed in the nerve bridge (Figures 7, 8). On day 9 and day 14, Schwann cell cords in the nerve bridge are still clearly visible and all Schwann cell cords have regenerating axons associated with them (Figures 7D–F,H). On day 9 and day 14, it appears that each Schwann cell cord in the nerve bridge has several regenerating axons associated with it (Figures 7D–F).

### Regenerating Axons Lose Their Directionality in the Nerve Bridge Owing to the Lack of Schwann Cell Guidance

On day 7 post-injury, Schwann cell cords have formed to guide the regenerating axons across the nerve bridge (Figure 6A). However, the Schwann cell cords formed in the nerve bridge are always not wide enough to guide all the regenerating axons across the nerve gap from the proximal nerve stump (Figure 6C). In the PLP-GFP mice on day 7, the diameter of Schwann cell cords in the nerve bridge could be easily observed by the GFP signal. Measuring the GFP signal showed that the diameter of the proximal nerve ends is about 1.01 ± 0.067 mm but the diameter of Schwann cell cords in the nerve bridge is only 0.41 ± 0.077 mm which is less than half of the diameter of the proximal nerve stump end (Figure 7G). The small diameter of Schwann cell cords in the nerve bridge resulted in more than half of regenerating axons apparently lacking guidance by Schwann cells (Figures 6A–C). On day 7, regenerating axons can be clearly classified into two populations in the nerve bridge due to the diameter of Schwann cell cords in the nerve bridge not being wide enough to guide all the regenerating axons across the nerve gap (Figure 6B). One population of axons growing inside the Schwann cell cords (indicated by white arrows in Figure 6B) will eventually cross the nerve gap and reach the distal nerve stump. The other population of axons (indicated by yellow arrows in Figure 6B), which are located outside of the Schwann cell cords are unable to grow further because they lack migrating Schwann cells at their front for guidance (Figures 6C,G–I). Indicated by their large diameter and the ball shape at the tip of the axons (Figures 6G–I), this population of axons are still in a state of random extension due to the lack of Schwann cell guidance. On day 7, they have formed at various angles relative to the nerve bridge, some axons near the epineurium can be seen that have turned their direction and have started to grow towards the proximal nerve stump (Figure 6B). With staining at the later timepoint of 9 days post-injury, we found that this population of axons have turned and grown along the outside of the proximal nerve trunk (indicated by white arrows in proximal nerve stump in Figure 7B). On day 14, we observed that more axons were growing along the outside of the proximal nerve trunk (Figures 8B,D). Thus, due to the lack of Schwann cell guidance in the front, a large population of regenerating axons from the proximal nerve stump have lost their directionality and failed to cross the injury site. This clearly showed that regenerating axons in the nerve bridge require Schwann cell guidance in order to cross the newly generated nerve bridge.

Previously, we also showed by whole-mount staining that there were regenerating axons extending along the outside the distal nerve stump on day 10, day 14 and day 90 post-transection (Dun and Parkinson, 2015). In this current study, we first observed regenerating axons growing along the outside the distal nerve in the PLP-GFP mice at 9 days post-injury (indicated by white arrows in distal nerve stump in Figure 7B), and this population of mis-directed axons is much easier to observe on day 14 (Figures 8B,E). Thus, some axons have followed the Schwann cell cords and crossed the nerve bridge, but they fail to enter into the distal nerve stump. Instead, they now misdirect their growth along the outside of distal nerve stump.

We further studied how migrating Schwann cells interact with misdirected regenerating axons on the surface of both the proximal and the distal nerve stumps on day 14. We always observed that the front of mis-directed axons are naked and there are no Schwann cells associated with them (Figures 8D,E). Further back on these mis-directed axons, Schwann cells could be observed associating with the axon bundles and these Schwann cells are often held by several axons (Figures 8D,E). In our previous study, these two populations of mis-directed axons extending along the outside of the nerve stumps were still observed on day 90 on the surface of both proximal and distal nerve stumps (Dun and Parkinson, 2015), suggesting that the neurons of mis-directed regenerating axons have still survived at this late timepoint, despite having not correctly re-innervated their targets.

**Figure 9:**
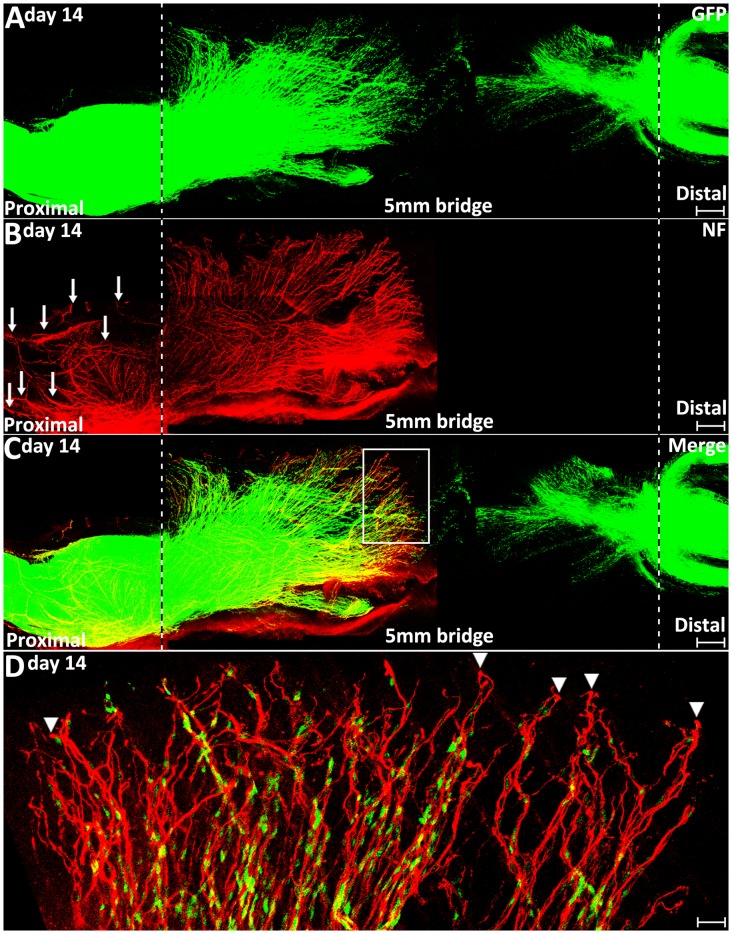
Axon regeneration and Schwann cell migration in a 5 mm sciatic nerve gap on day 14 post-injury. **(A–C)**. Whole nerve preparation at 14 days post-injury shows regenerating axons and migrating Schwann cells in a 5 mm sciatic nerve gap of PLP-GFP mice. The cut ends of both proximal and distal nerve are indicated by dashed lines. Arrows in **(B)** indicate regenerating axons growing along the outside of the proximal nerve stump. **(D)** Higher magnification image from the box area in **(C)** shows regenerating axons proceeding in front of migrating Schwann cells in the proximal nerve end on day 14 post-injury. Arrowheads indicate the ball shape at the tips of regenerating axons. Scale bars in **(A–C)** 250 μm. Scale bar in **(D)** 30 μm.

In clinical peripheral nerve repair, a nerve graft or conduit repair is required for the treatment of nerve gaps equal to or greater than 5 mm in length (Deumens et al., 2010; Ray and Mackinnon, 2010; Daly et al., 2012). Schwann cell cords are unable to form and regenerating axons from proximal nerve are unable to cross a 5 mm nerve gap and enter into the distal nerve stump without a nerve graft or conduit repair. Finally, we generated 5 mm length sciatic nerve gaps by removing a piece of nerve to study how migrating Schwann cells interact with regenerating axons in a 5 mm sciatic nerve gap in the PLP-GFP mice on day 14. We found the Schwann cell cords were never formed across a 5 mm mouse sciatic nerve gap (Figure 9A). In the proximal nerve stump, even on day 14, regenerating axons are still seen extending in front of Schwann cells migrating from the proximal stump. Several regenerating axons form bundles and hold Schwann cells and a ball shape is often seen at the tips of regenerating axons. Regenerating axons appear more scattered and form a fan shape due to the lack of their directionality (Figures 9B–D). In the distal nerve end, Schwann cells still form chains and migrate out from the distal nerve end, but the majority of them are not facing towards the proximal nerve ends (Figure 9A). These observations further suggest that the lack of Schwann cell guidance is the primary reason resulting in axon mis-targeting in the nerve bridge.

## Discussion

Schwann cells are the peripheral nerve glia and the injury-induced Schwann cell plasticity is essential for the success of peripheral nerve regeneration and tissue repair (Lopez-Verrilli et al., 2013; Jessen and Mirsky, 2016; Carr and Johnston, 2017). Current evidences suggest that peripheral nerve-associated Schwann cells possess the capacity to promote repair in multiple tissue including peripheral nerve gap bridging, skin wound healing and digit tip regeneration (Johnston et al., 2016; Carr and Johnston, 2017; Parfejevs et al., 2018). In this study, we showed that the PLP-GFP mouse is an excellent mouse model to visualize *in vivo* Schwann cell migration after peripheral nerve transection injury. Several other genetic approaches have been used to label Schwann cells with fluorescent proteins in the mouse such as S100-GFP (Zuo et al., 2004), S100-BFP, S100-RFP (Hirrlinger et al., 2005) and Sox10-Venus (Hirrlinger et al., 2005). Among all of them, the PLP-GFP and S100-GFP mice are the best characterized mouse models to reveal *in vivo* Schwann cell migration during peripheral nerve regeneration (Hayashi et al., 2007; Tomita et al., 2009; Whitlock et al., 2010; Cattin et al., 2015; Stierli et al., 2018; Zigmond and Echevarria, 2019).

The basic behavior of *in vivo* axon regeneration and Schwann cell migration after peripheral nerve transection injury has been studied by Torigoe et al. (1996) using the film model. In the film model, the transected mouse proximal peroneal nerve was sandwiched between two thin plastic fluorine resin films. A very thin layer of regenerating tissue could be formed between two films. Therefore, tissue sectioning was not needed for subsequent analysis in this model. Using this method, Torigoe et al. (1996) analyzed axon regeneration on the film in the early phase of regeneration up to 6 days. However, a nerve bridge is unable to form in the film model, therefore the film model cannot mimic the full *in vivo* nerve bridge microenvironment for axon regeneration and Schwann cell migration. In our study, combining our whole-mount staining method with the use of the PLP-GFP mouse model, we are able to study the basic behavior and the time course of *in vivo* axon regeneration, Schwann cell migration and Schwann cell-axon interaction in the nerve bridge. We were able to provide much clearer images covering the whole field of the injury site of a transected mouse sciatic nerve at different time points and to analyze the regeneration events in the mouse sciatic nerve bridge. Using this methodology, we not only demonstrated that Schwann cells play a crucial role in guiding axon regeneration across the nerve gap, but also revealed that the lack of Schwann cell guidance in the nerve gap is the apparent reason for axons misdirection during regeneration.

Since Ramón y Cajal’s initial observations, there have been intensive debates about whether axons regenerate randomly in the nerve gap without the guidance of Schwann cells or they are actually guided by Schwann cells (Lobato, 2008). Questions have also been raised whether Schwann cells act as a leader or a follower during the period of axons navigation across the peripheral nerve gap (Keynes, 1987). Using the film model, Torigoe et al. (1996) analyzed Schwann cell migration on the film after S-100 antibody staining. In their study, Schwann cells start to appear on day 3 near the transected nerve stump and the number of Schwann cells gradually increased on day 4. At this stage, Schwann cells showed a preference for axonal surfaces as a migrating pathway over any other environmental structure (Torigoe et al., 1996). The expression of adhesion molecules on the axonal surface has been suggested as the primary reason that Schwann cells migrate along the regenerating axons (Torigoe et al., 1996). In this study, we also showed that regenerating axons proceed in front of the migrating Schwann cells on day 4 post-injury. There are no Schwann cells associated with the front of regenerating axons and these axons have been previously described by Cajal as “naked axons” (Lobato, 2008). Thus, Torigoe et al.’s (1996) observation together with our finding have provided evidence that axons regenerate randomly when there are no preceding Schwann cells.

In response to peripheral nerve injury, neurons rapidly activate a remarkable intrinsic program to regenerate (Chen et al., 2007; Rishal and Fainzilber, 2010; Jessen and Mirsky, 2016). In mouse, regenerating neurites sprout from the first node of Ranvier proximal to the site of nerve injury just 3 h after axotomy and they pass the tips of the proximal nerve 6 h after axotomy (Torigoe et al., 1996). Following a transection injury, Schwann cells near the injury site of the proximal nerve stump and all Schwann cells in the distal nerve stumps undergo a rapid process of dedifferentiation and proliferation (Jessen and Mirsky, 2016). These processes take 2–3 days to complete before they can migrate, this could be the primary reason that the start of Schwann cell migration is much later than the start of axon extension.

Torigoe et al. (1996) showed in the film model that migrating Schwann cells proceed in front of regenerating axons on day 5 following mouse peroneal nerve transection injury (Torigoe et al., 1996). In this study, we also observed that migrating Schwann cells in the proximal nerve stump start to proceed in front of regenerating axons on day 5. They use regenerating axons as a substrate to migrate before day 5 following mouse sciatic nerve transection injury. Thus, migratory Schwann cells in the proximal nerve stump initially follow the regenerating axons and migrate into the nerve bridge, they are followers of regenerating axons before day 5 of regeneration. From day 5 onwards, migrating Schwann cells locate in the front of regenerating axons and become leaders to direct axon regeneration across the nerve gap.

Migration of Schwann cells into the nerve gap after peripheral nerve transection injury is essential for successful peripheral nerve regeneration. They form Schwann cell cords in the nerve gap to guide axons from the proximal nerve stump into the distal nerve stump. Using the film model, Torigoe et al. (1996) revealed that the speed of axon extension has two phases, an initial slow phase (77 μm/day) when axons are naked followed by a faster phase (283 μm/day) when Schwann cell migrate into the front. The appearance of migrating Schwann cells to the regenerating edge coincides with the onset of the second phase of axon growth, therefore migrating Schwann cells appear to be responsible for the acceleration of axonal growth in the second phase (Torigoe et al., 1996). In our study, we demonstrated that random extension axons grow with both a low speed (85.7 μm/day) combined with a lack of directionality without Schwann cells at the front. Axons increase their speed to 433.1 μm/day when there are migrating Schwann cells acting as substrates for extension. In comparison to the film model, we observed a slightly faster speed of axon regeneration in both phases following injury than Torigoe et al.’s (1996) measurement; one explanation for this faster regeneration rate may be that the nerve bridge is correctly formed in our research model.

The morphology of migrating Schwann cells have been largely studied using *in vitro* culture conditions (Wang et al., 2012). In vitro, Schwann cells have a bipolar shape and often only have one migrating process in the front. Interestingly, we have observed *in vivo* that the pioneer cells often have two or three leading processes during migration, which indicates that these cells seem to be detecting environmental signals suitable for migration. Previous studies have suggested that fibrin deposits are the substrates for Schwann cell migration in the nerve bridge (Williams et al., 1983; Schröder et al., 1993). However, a recent report showed that Schwann cells use newly formed blood vessels as a substrate to migrate upon Cattin et al. (2015). In agreement with Torigoe et al.’s (1996) finding, our observation also showed that migrating Schwann cells in the proximal nerve stump use regenerating axons as a substrate to migrate upon before day 5. These observations showed that Schwann cells may use multiple sources of substrate for migration in the nerve bridge.

In our experiments, we did not remove the epineurium for the whole-mount staining in order to preserve the full pattern of regenerating axons around the nerve bridge area. The epineurium prevents antibody penetration. Therefore, the neurofilament antibody staining will only reveal regenerating axons inside the nerve bridge as well as regenerating axons growing on the outer surface of both nerve stumps (Dun and Parkinson, 2015). Our observations showed that there are two populations of mis-guided regenerating axons growing along the surface of both the proximal and the distal nerve stumps. One population of axons leave the proximal nerve, turn back and then grow back along the outside of the proximal nerve stump, presumably due to the lack of guidance by migrating Schwann cells. We showed by the GFP signal in the PLP-GFP mice that Schwann cells cords in the nerve bridge appear apparently not wide enough for all the regenerating axons that are required to cross the newly formed nerve bridge at this timepoint. Previously, Williams et al. (1983) also showed in a silicone tube nerve conduit apparatus that the nerve bridge has a conical shape and the diameter of the nerve bridge is always narrower than both the nerve ends. This conical bridge shape has also been reported in the studies of peripheral nerve gap repaired with modern biodegradable nerve guidance conduits (Belkas et al., 2004; Moore et al., 2009; Sun et al., 2010). Schwann cell cords are the key component in the nerve bridge to guide axon regeneration. Thus, our observation in the PLP-GFP mice has identified the most important reason for the misdirection of regenerating axons in the nerve bridge, which is that the area of Schwann cell cords in the nerve bridge is not wide enough to guide all the regenerating axons from the proximal nerve stump across the nerve bridge. We believe that providing enough Schwann cells as a substrate to guide all the regenerating axons cross the nerve gap will be one of the important strategies to improve functional recovery after peripheral nerve injury.

## Data Availability Statement

All datasets generated for this study are included in the article.

## Ethics Statement

The animal study was reviewed and approved by Plymouth University Animal Welfare and Ethical Review Board.

## Author Contributions

XD designed the research. BC, QC and XD performed experiments and analyzed the data. XD and DP wrote the article.

## Conflict of Interest

The authors declare that the research was conducted in the absence of any commercial or financial relationships that could be construed as a potential conflict of interest.
